# Fabrication of photoactive heterostructures based on quantum dots decorated with Au nanoparticles

**DOI:** 10.1080/14686996.2016.1153939

**Published:** 2016-04-12

**Authors:** Elisabetta Fanizza, Carmine Urso, R. Maria Iacobazzi, Nicoletta Depalo, Michela Corricelli, Annamaria Panniello, Angela Agostiano, Nunzio Denora, Valentino Laquintana, Marinella Striccoli, M. Lucia Curri

**Affiliations:** ^a^ Dipartimento di Chimica, Università degli Studi di Bari, Via Orabona 4, 70126Bari, Italy; ^b^ Istituto per i Processi Chimico Fisici IPCF Consiglio Nazionale delle Ricerche CNR, Via Orabona 4, 70126Bari, Italy; ^c^ Dipartimento di Farmacia – Scienze del Farmaco, Università degli Studi di Bari, Via Orabona 4, 70126Bari, Italy; ^d^ Istituto tumori IRCCS Giovanni Paolo II, Bari, Italy

**Keywords:** Quantum dots, metal nanoparticles, plasmonic luminescent nanostructures, multimodal bioimaging, 10 Engineering and Structural materials

## Abstract

Silica based multifunctional heterostructures, exhibiting near infrared (NIR) absorption (650–1200 nm) and luminescence in the visible region, represent innovative nanosystems useful for diagnostic or theranostic applications. Herein, colloidal synthetic procedures are applied to design a photoactive multifunctional nanosystem. Luminescent silica (SiO_2_) coated quantum dots (QDs) have been used as versatile nanoplatforms to assemble on their surface gold (Au) seeds, further grown into Au spackled structures. The synthesized nanostructures combine the QD emission in the visible region, and, concomitantly, the distinctive NIR absorption of Au nanodomains. The possibility of having multiple QDs in a single heterostructure, the SiO_2_ shell thickness, and the extent of Au deposition onto SiO_2_ surface have been carefully controlled. The work shows that a single QD entrapped in 16 nm thick SiO_2_ shell, coated with Au speckles, represents the most suitable geometry to preserve the QD emission in the visible region and to generate NIR absorption from metal NPs. The resulting architectures present a biomedical potential as an effective optical multimodal probes and as promising therapeutic agents due to the Au NP mediated photothermal effect.

## Graphical abstract

**Figure:**
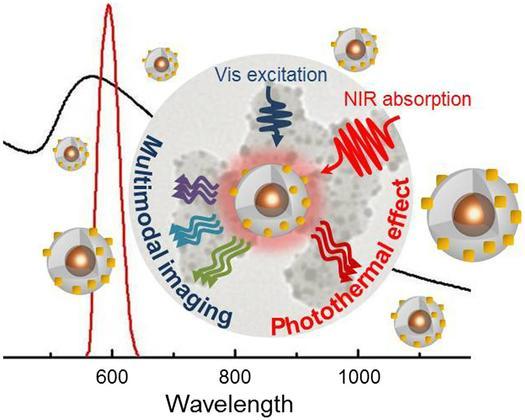

## 1. Introduction

Engineered photoactive nanomaterials represent a great promise for the advance of the next generation of nanomedicine tools, such as tags for imaging and early-stage diagnostic and therapeutic monitoring of diseases [1–3]. Current research efforts are focused on the design, development and application of multifunctional nanoparticles (NPs) that simultaneously provide cancer diagnostics and therapy, such as nanostructures able to specifically locate and selectively destroy cancer cells.

Efforts to create these multicomponent nanostructures have largely been driven by their specific chemical and physical properties and by their expected functionality, which make them advantageous in applications otherwise inaccessible by their single components.

Plasmonic NPs, exhibiting absorption in the visible and near infrared (vis-NIR) window (700–1200 nm), are clinically important owing to the low extinction coefficients of water, tissue, and blood in this region of the electromagnetic spectrum. Several gold (Au) based NIR absorbing materials have recently been developed, such as Au NPs of different shapes (nanorods, nanocages, agglomerates, hollow, pentagons, and large prisms) and Au composites (SiO_2_@Au nanoshells and superparamagnetic iron oxide nanoshells) [4–8], which can be investigated by different imaging modalities, such as confocal microscopy, photoacoustic tomography, computed tomography, and dark-field light scattering imaging, and can be used to deliver localized photothermal therapy [9–11].

Heterostructures, that combine the NIR absorption of plasmonic NPs with supplementary functionalities, such as photoluminescence or magnetism, appear attractive for application in nanomedicine [1–3, 6, 8, 10–13]. Such nanostructures have potential as versatile multimodal bioimaging agents and efficient therapeutic tools by photothermal ablation, and hence for a simultaneous early stage detection and non-invasive treatment of various diseases, especially of cancer [1–3].

Here, the integration of NIR absorbing and luminescent materials into a single architecture with a size of a few tens of nanometers is presented. The all-inorganic heterostructure proposed in this work is based on plasmonic Au NPs and fluorescent CdSe@ZnS quantum dots (QDs), more stable with respect to organic dyes. Only a few reports have so far dealt with this type of heterostructure [15–18], applied as contrast agents for plasmon and fluorescence-based imaging and detection, with potential for image-guided photoablation therapy.

However the fabrication of this class of heterostructures imposes the control of the interaction between the plasmonic and luminescent domains, which depends on the mutual distance between plasmonic and fluorescent particles down to the nanometer level. It is known that QD emission intensity can be quenched or, alternatively, enhanced by carefully tailoring the distance from the plasmonic moiety [15–18]. In addition the plasmonic component must be properly designed in order to have absorption in the NIR region.

Here, a colloidal silica shell (SiO_2_) has been selected as a robust scaffold and a versatile nanoplatform for combining the distinct spectral features of the fluorescent and plasmonic component [8]. By means of a multistep synthetic approach, QDs have been coated with a silica shell that has been further functionalized with Au nanodomains. The tunable thickness of the SiO_2_ represents an adjustable spacer to properly modify the distance between metal NPs and luminescent QDs, and, hence, to control their interaction.

Recently, it has been reported [19] that SiO_2_ NPs, coated with an Au shell, resulted in nanostructures with strong absorption and scattering ranging from the visible to near-IR region, depending on the core/shell size ratio. However most works concern Au nanoshells grown onto SiO_2_ NPs of large diameter (between 80 and 150 nm), that show restricted application *in vivo* due to the limited diffusion in tissue of such large particles [19]. Few studies of NIR absorbing plasmonic NPs with sizes below this range have been reported [19]. The nanostructure proposed here, purposely designed with a size of a few tens of nanometers, can be envisaged to provide a significant contribution to this perspective.

A comprehensive optical (UV-vis absorbance, photoluminescence and infrared spectroscopy) and structural (transmission electron microscopy) investigation of the prepared structures has been performed in order to define the experimental conditions to achieve the most suited architecture able to combine NIR absorption and luminescence in the visible range. The cytotoxicity of nanostructures has been carefully evaluated after each fabrication step by means of *in vitro* studies on rat C6 glioma cells line, in order to assess their impact in a biological environment and thus makes such a multifunctional structure, potential for simultaneous multimodal imaging and therapy [15]. In addition, both SiO_2_ and Au, generally regarded as safe biomaterials [20–25], provide surface anchoring points for efficient biomolecular conjugation for targeting applications [26–31].

## 2. Materials and methods

### 2.1. Chemicals

The CdSe@ZnS QD synthesis and surface processing were achieved through the following reagents: cadmium oxide (99.5% powder, CdO), trioctylphosphine oxide (99%, TOPO), t-butylphosphonic acid (98%, tBuPOH), hexadecylamine (technical grade 90%, HDA, Fluka, Milan, Italy), tributylphosphine (97%, TBP), selenium (99.99% powder, Se), sulfur (99.9999% powder, S), trioctylphosphine (90%, TOP), diethylzinc solution (1 M in heptane, Et_2_Zn), hexamethyldisilathiane (HMST). The SiO_2_ shell growth was carried out by: tetraethyl orthosilicate (98% d = 0.934 g ^ml–1^, TEOS), aqueous ammonia (d = 0.900 g ^ml–1^, NH_4_OH), 5 polyoxyethylene nonylphenylether (igepal CO-520, Mn = 441), and surface functionalization with (3-aminopropyl)triethoxysilane (97%, APS), sodium hydroxide (NaOH), tetrakis(hydroxymethyl) phosphonium chloride (THPC, 80% in H_2_O), gold(III) chloride hydrate (HAuCl_4_•H_2_O, 99.9%), potassium carbonate (K_2_CO_3_ 99.8%), formaldehyde. For the ninhydrin test, ninhydrin and 2,6-lutidine (98+%, Alfa Aesar, Milan, Italy) were used. All chemicals, purchased from Sigma Aldrich (Milan, Italy) if not specified otherwise, were used without purification. All solvents, namely methanol, ethanol, chloroform, cyclohexane were of the highest purity available.

### 2.2. Synthesis of SiO_2_ coated CdSe@ZnS QDs (QD@SiO_2_)

The silica shell growth onto the ‘as-synthesized’ CdSe@ZnS QDs (see Supplementary Data for experimental details) was carried out by using a water-in-oil microemulsion approach [32–36]. Sequentially, 350 μl of IGEPAL CO-520, 200 μl of NH_4_OH and a varied volume of TEOS (in the range of 20 to 50 μl) were injected to 6 ml of a cyclohexane solution of QDs (1.5·10^−6^ M). The solution was then kept under vigorous stirring and controlled temperature 28°C for 18 h.

Methanol was added to disrupt the micelle and the QD@SiO_2_ NPs were collected by centrifugation at 7800 g for 20 min (Beckman J2-21, Rodano (MI), Italy). Repeated cycles of NP dispersion in ethanol and centrifugation were carried out to completely remove surfactant residuals. The prepared NPs were finally dispersed in 4 ml of ethanol. The QD@SiO_2_ concentration in solution was nearly 10^15^ NPs in 4 ml of ethanol, and was calculated by using the method described in the Supplementary Material.

### 2.3. Surface functionalization with amine groups and characterization of QD@SiO_2_ nanoparticles

Surface functionalization reactions of QD@SiO_2_ NPs with amine groups were carried out adding equal volume of ammonia solution and APS, 20, 80 or 120 μl, respectively, to 2 ml of NP suspension prepared by diluting 400 μl of QD@SiO_2_ samples with ethanol. The final suspension was stirred overnight at 28°C and the functionalized NPs were collected by centrifugation and cleaned up from unreacted reagents by repeated cycles of centrifugation/redispersion in ethanol.

The ninhydrin test, which allows an effective characterization and quantitative analysis of primary amine (NH_2_) groups in different types of system, including biomolecules [37], was here modified to investigate the functionalization step of QD@SiO_2_ and to quantify the yield of surface functionalization. A ninhydrin solution was preliminary prepared by dissolving 110 mg of ninhydrin in 16 ml of ethanol (0.68% w/v) and adding 4 ml of 2,6-lutidine.

Preliminary calibration experiments were performed by adding an excess of ninhydrin solution in ethanol to APS standard solutions ranging from 50 μM to 15 mM. Upon heating in a thermostatic bath at 90°C for 5 min, in the presence of NH_2_ groups, the solution color changes from pale yellow (free ninhydrin) to blue due to the formation of Ruhemann’s Blue byproduct, thus providing an evidence of the presence of the amine groups. The absorption intensity at 570 nm, characteristic of the Ruhemann’s Blue byproduct, monitored by UV-vis absorbance spectroscopy, allows us to plot a calibration line and to determine the extinction coefficient (ε_570 nm_ = 49 l mol^–1^ cm).

The addition of an excess of ninhydrin solution to the NH_2_-functionalized NP suspension and the spectrophotometric characterization were carried out. In particular, 1.9 ml of ninhydrin solution (3·10^−2^ M) were added to 0.1 ml of NPs collected after the functionalization reaction. The suspension was then warmed up at 90°C for 5 min. The change in the QD@SiO_2_ suspension color from orange to blue (color of the Ruhemann’s Blue assay byproduct) accounts for the presence of NH_2_ groups, and the absorbance intensity measured at 570 nm, characteristic of the Ruhemann’s byproduct, was used to titrate the amount of amino groups in each sample. The absorbance intensity at 570 nm of the NH_2_-functionalized QD@SiO_2_ samples was corrected by considering the contribution of the bare QD@SiO_2_ to the absorption.

### 2.4. Growth of Au nanoparticles onto QD@SiO_2_

The growth of Au NPs onto the silica surface was carried out following a reported method [38, 39] based on the three following steps: synthesis of 2 nm Au seeds, their assembly onto NH_2_-functionalized QD@SiO_2_ and finally the Au growth [38]. Au NPs were synthesized as reported elsewhere: 2 ml of 30 mM HAuCl_4_ solution in milliQ H_2_O was added in a three neck flask containing the reaction mixture based on 0.1 M NH_4_OH and 0.17 M THPC solution, in a total volume of 48 ml of milliQ H_2_O [38]. The solution was stirred at room temperature for about 30 min, meanwhile it turned from a pale yellow color of the Au salt, to a brown tint, corresponding to formation of small Au NPs.

The decoration of the NH_2_-functionalized QD@SiO_2_ with Au seeds was carried out by simply mixing 5 ml of the Au seed solution to 0.5 ml of NH_2_-functionalized NPs under stirring for about 2 h. The beads were recovered by centrifugation at 7800 g, which allowed removal of excessive seeds, as well. The final precipitate was dissolved in 0.5 ml of milliQ H_2_O.

The Au seeds decorating the silica surface were then used to grow Au nanoislands by addition of 4 ml of the Au salt solution in milliQ H_2_O, containing also K_2_CO_3_ and HAuCl_4_. Different K_2_CO_3_/HAuCl_4_ (concentration of K_2_CO_3_ and HAuCl_4_ equal to 1.8 mM/0.4 mM, 0.9 mM/0.2 mM and 0.2 mM/0.05 mM in milliQ H_2_O) were tested to study the influence of the Au salt concentration on the final Au nanostructure size. After about 30 min stirring at room temperature, 10 μl of formaldehyde solution 0.3 M in milliQ H_2_O was swiftly added. The reaction typically completed in 30 min.

The Au seed decoration process and subsequent growth of Au nanodomains onto the luminescent QD@SiO_2_ were monitored by transmission electron microscopy (TEM) and optical absorbance and photoluminescence (PL).

### 2.5. Optical characterization

UV-vis absorption spectra were recorded with a Cary 5000 (Varian, Leini, (TO), Italy) UV/Vis/NIR spectrophotometer. PL spectra were recorded by using a Fluorolog 3 spectrofluorimeter (HORIBA Jobin-Yvon, Edison, New Jersey, USA), equipped with double grating excitation and emission monochromators. All optical measurements were performed at room temperature on samples obtained directly from synthesis without any size sorting treatment. Transient-PL measurements were performed using the time-correlated single photon counting (TCSPC) technique, with a FluoroHub (HORIBA Jobin-Yvon). The samples were excited at 375 nm by a picosecond laser diode (NanoLED 375L) emitting τ≈80 ps pulses at a 1 MHz repetition rate. The PL signals were dispersed by a double grating monochromator and detected by a picosecond photon counter (TBX ps Photon Detection Module, HORIBA Jobin-Yvon, Edison, New Jersey, USA). The temporal resolution of the experimental setup was ≈200 ps.

Relative PL quantum yield (QY) was measured by dispersing the samples in dimethylsulfoxide (DMSO) and using cumarine 153 in DMSO as a standard (absolute QY in DMSO 75%) due to its spectroscopic properties (emission centered at 530 nm and excitation spectra in the range of 370–450 nm, thus suitably matching the emission spectrum range of QDs). The excitation wavelength used for the PL QY measurements was 395 nm.

The absorbance of the Ruhemann’s byproduct at 570 nm was determined using a Perkin Elmer (Monza, MI, Italy) 2030 multilabel reader Victor TM X3.

### 2.6. Transmission electron microscope

A JEOL JEM 1011 electron microscope operating at 100 kV and equipped with a high resolution CCD camera was used for TEM analysis. The sample was prepared by casting a drop of suitably diluted solution of NPs and QDs on the surface of a carbon deposited copper grid. Statistical analysis of the size (NP average size and size distribution) of the samples has been performed by use of a freeware image analysis program. In particular the average NP size and the percentage relative standard deviation (σ%) have been calculated for each sample. Such final statistical determination provides information on the NP size distribution. Its value is based on the spread of size compared to the average value and is expressed as a percentage.

### 2.7. Particle size, size distribution and surface charge

Hydrodynamic diameter (size), size distribution and colloidal stability of the nanostructures, before and after the growth of Au nanodomains on luminescent silica coated NPs, were detected using a Zetasizer Nano ZS, Malvern Instruments Ltd, Worcestershire, UK (DTS 5.00). In particular, size and size distribution were determined by means of dynamic light scattering (DLS) after sample dilution in demineralized water. Hydrodynamic diameter is reported by number and size distribution is described in terms of polydispersity index (PDI). The ζ-potential measurements, i.e. the surface charges, were carried out by using a laser Doppler velocimetry (LDV) after sample dilution in KCl aqueous solution (1 mM). All reported data are presented as mean values ± standard deviation of three replicates.

### 2.8. *In vitro* cytotoxicity of NPs and QDs

Cytotoxicity assays were carried out against rat C6 glioma cells. This cell line was maintained at 37°C in a humidified incubator containing 5% CO_2_ in Dulbecco’s modified Eagle’s medium (Lonza, Caravaggio (BG), Italy) nutrient supplemented with 10% heat inactivated fetal bovine serum, 2 mM L-glutamine, 100 U ml^–1^ penicillin and 100 μg ml^–1^ streptomycin. The cell viability was determined using the 3-(4,5-dimethylthiazol-2-yl)-2,5-diphenyl-tetrazolium bromide (MTT) assay. Cells were dispensed into 96-well microtiter plates at a density of 5000 cells/well. Following overnight incubation, cells were treated with the different compounds, QD@SiO_2_, APS-functionalized QD@SiO_2_ and Au speckled QD@SiO_2_, at different concentrations (ranging from 0.1 to 0.001 mg ml^–1^). Then the plates were incubated at 37°C for 72 h. 10 μl of 0.5% w/v MTT were further added to each well and the plates were incubated for an additional 4 h at 37°C. Finally the cells were lysed by addition of 100 μl of 1:1 v/v DMSO:EtOH solution. The absorbance at 570 nm was measured by using a Perkin Elmer 2030 multilabel reader Victor TM X3. Each test was performed in triplicate in three separate experiments. The result is expressed as the IC_50_ value that is the concentration necessary for 50% inhibition.

## 3. Results and discussion

The work aims at preparing nanostructures, with size of a few tens of nanometers, having visible light emission and NIR absorption, as potential colloidal systems for multimodal imaging and photothermal therapy.

**Scheme 1.**
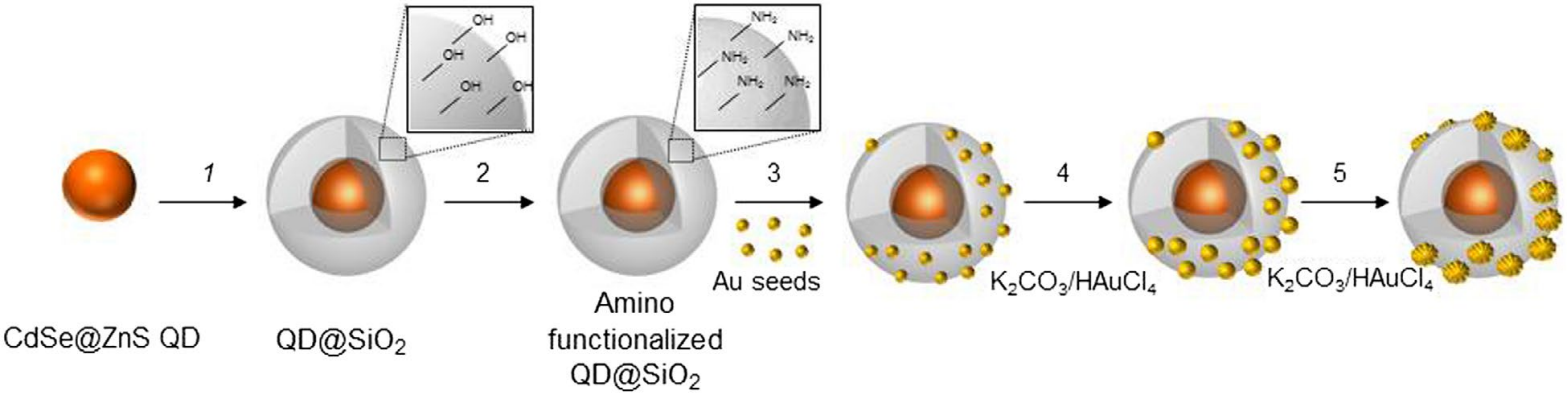
General scheme for the QD functionalization process leading to SiO2 shell growth (step 1), functionalization with amine groups upon reaction with (3-aminopropyl)triethoxysilane (step 2) and subsequent assembly of Au seeds and progressive formation of Au NPs upon the addition of K2CO3 and HAuCl4 (steps 3, 4, 5).

Scheme 1 reports the fabrication routes designed to accomplish the proposed multifunctional nanostructures, which, in particular, combines CdSe@ZnS QDs as inorganic fluorophore [29–31], with emission in the visible light region, and Au metal NPs, whose plasmon absorption can be tuned up to the NIR region of the electromagnetic spectrum [19, 38–41].

Scheme 1. General scheme for the QD functionalization process leading to SiO_2_ shell growth (step 1), functionalization with amine groups upon reaction with (3-aminopropyl)triethoxysilane (step 2) and subsequent assembly of Au seeds and progressive formation of Au NPs upon the addition of K_2_CO_3_ and HAuCl_4_ (steps 3, 4, 5).

The presence of an SiO_2_ shell allows the combination of the distinct spectral features of fluorescent quantum dots and the metal NPs, by encapsulating one component inside and binding the other ones on its surface. Thanks to the control on its thickness, it represents an adjustable spacer to modulate the distance between metal NPs and luminescent QDs, circumventing significant QD quenching phenomena and thus providing a detectable fluorescence. In addition the dielectric–metal interface enables the geometric ‘tunability’ of the plasmon resonances of Au NP, inducing size dependent broad absorption, ranging from visible to NIR region.

Furthermore, growth of a silica shell onto Cd based QDs is an efficient method to reduce their toxicity and impart them dispersibility in aqueous media, thus enabling development of safe systems for biomedical applications [23–25]. Such a surface also offers a versatile platform not only for further functionalization with NIR absorbent domain, but also for further bioconjugation with specific biomolecules [32–36].

The overall multistep synthetic process has been designed to provide a single multifunctional heterostructure dispersible and stable in aqueous media with a rational control of the optical properties and interaction between the photoactive components.

### 3.1. Spectroscopic and morphological characterization of QD@SiO_2_

*As*-*synthesized* luminescent organic-capped CdSe@ZnS QDs (see Supplementary Material Figure SI1) have been coated with a silica shell by following a well-established water-in-oil microemulsion synthetic route, which has been demonstrated as a suitable and reliable strategy for hydrophobic NPs in the range of a few nanometers [32–41]. This synthetic strategy limits the size of the final core/shell structure below 100 nm in diameter, and hence produces NPs that may more efficiently penetrate the cell membrane [19].

In a typical experiment, QDs are dispersed in cyclohexane and Igepal CO-520 as surfactants (which are non-toxic, cheap and easy to handle), and ammonia solution and TEOS are added to the reaction flask and stirred at room temperature. The TEOS concentration is varied, keeping constant all the other parameters and the final core/shell heterostructure morphology and optical properties are investigated.

**Figure 1.**
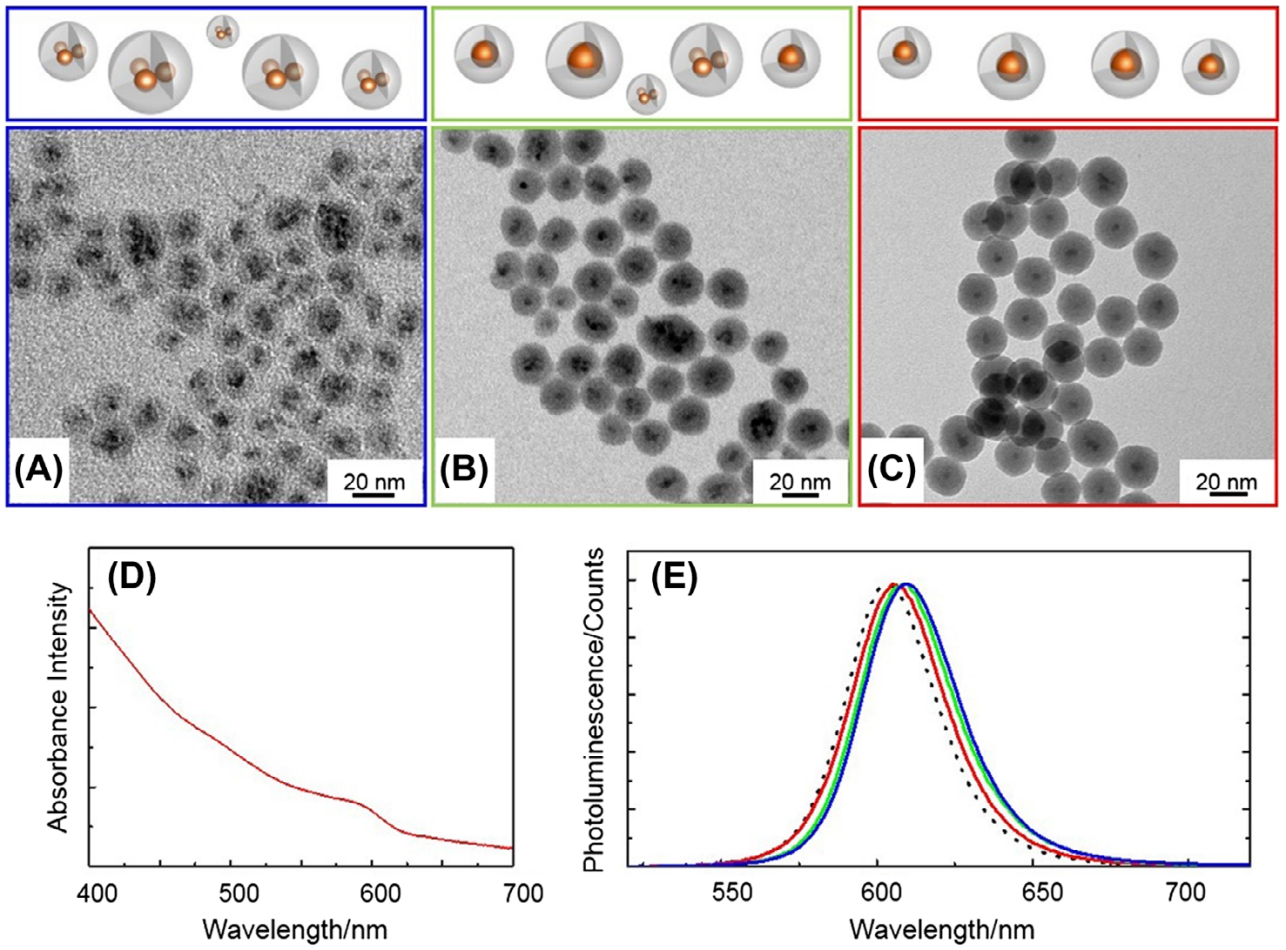
TEM images (A–C), UV-vis absorbance (D) and photoluminescence spectra (E) of QD@SiO_2_ prepared at a QD concentration of 10^−6^ M in cyclohexane, IGEPAL CO520 (350 μl), NH_4_OH (200 μl), by adding 20 μl (A, E blue line), 30 μl (B, E green line) and 50 μl (C, D, E red line) of TEOS. PL spectrum of bare QDs in chloroform (E, dashed line).

Figure 1 shows TEM images (A–C), UV-vis absorbance (D) and PL (E) spectra of the QD@SiO_2_ sample prepared at 10^−6^ M QD concentration (in cyclohexane) with TEOS amounts of 20, 30, and 50 μl (resulting in the samples QD@SiO_2__20, QD@SiO_2__30 and QD@SiO_2__50 respectively).

The TEM characterization highlights that nanostructure morphology and silica shell thickness can be tuned by means of a careful control of the amount of TEOS in the reaction. At 20 μl of TEOS, the resulting nanostructures (Figure 1(A)) show a large fraction of multiple QD in the cores (nearly 92% multicores versus 8% of single core NPs), a size of nearly 16 nm, a broad size distribution (σ% = 20%), and a poorly uniform shell (thickness is 6 nm, σ% = 17%). Multiple QD cores and single QD core silica coated core-shell nanostructures will be hereafter called QD_n_@SiO_2_ and QD@SiO_2_, respectively.

By increasing the TEOS volume from 20 to 50 μl, a thicker and more uniform silica shell can be obtained (Figure 1(B) and (C)). The addition of 30 μl of TEOS in the reaction resulted in a decreased core multiplicity, with a higher incidence (58%) of single core NPs (QD@SiO_2_ NPs, Figure 1(B)). Meanwhile an increase in the size of the NPs (23 nm, σ_%_ = 20%) and shell thickness (nearly 10 nm, σ_%_ = 17%) occurs.

Use of a larger volume (50 μl) of TEOS (Figure 1(C)) has produced a highly monodisperse single core QD@SiO_2_ NPs (size 38 nm, σ_%_ = 8%), very uniform in shape, with a silica shell thickness of 16 nm (σ_%_ = 13%). The very thin silica shell and the concomitant occurrence of NPs with multiple QD cores (Figure 1(A)), observed at low TEOS content, can be explained considering that the limited amount of hydrolyzed TEOS is not sufficient to uniformly coat the QD surface. In fact, at low TEOS content, assemblies of QDs rather than single QDs are incorporated in the aqueous domains of the micelle, finally resulting in multiple core structures. By increasing up to 50 μl the amount of TEOS, the amount of silica precursor becomes sufficient to coat single QDs, thus resulting in a higher yield of single core NPs. In addition, such an amount of silica precursor is abundant enough to allow the growth of a thicker shell, as shown in Figure 1(B) and (C). Nevertheless such a TEOS content is still below the amount that results in homogeneous nucleation of empty SiO_2_ NPs [42, 43].

As potential optical probes, the emission properties of the nanostructures have also been investigated. First, a slight red shift of the PL peak is observed in the spectra of the QD@SiO_2_ samples (Figure 1(E) blue, green and red line) compared to that of the bare QDs (Figure 1(E) dashed line), the extent of which is a function of the volume of TEOS precursor used, as the lower the amount of TEOS, the larger the red shift of the emission peak. The shift with respect to native QDs can be definitely associated to the presence of the silica shell while the shift moving from QD@SiO_2__20 to QD@SiO_2__50 samples (Figure 1(A)–(C)) can be ascribed to collective energy transfer phenomena among the QDs trapped in the same SiO_2_ matrix.

**Figure 2.**
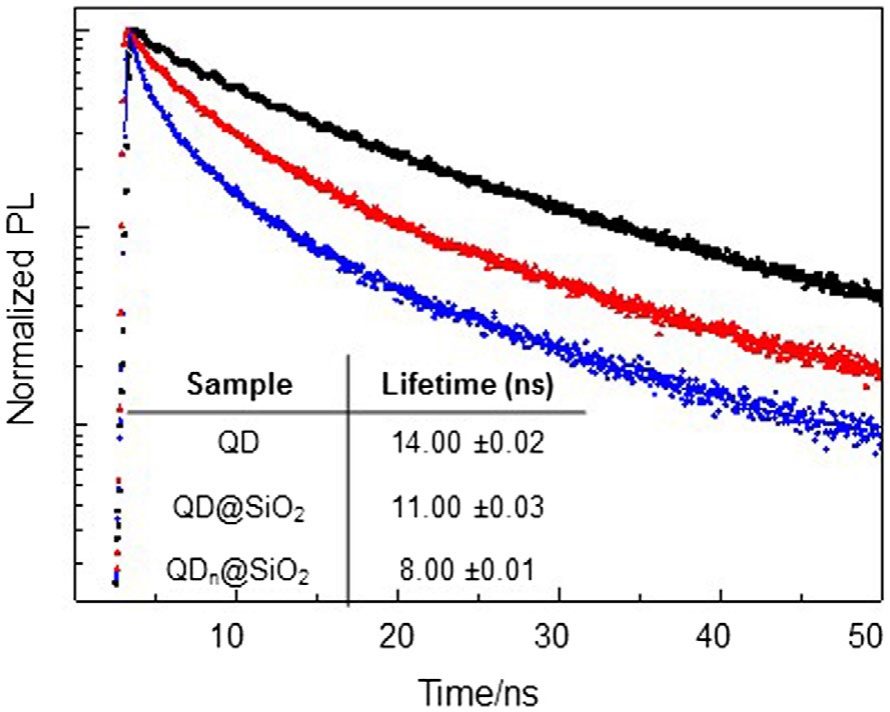
Time-resolved fluorescence intensity decays (A) and average lifetime (B) of QD (black line, QD@SiO_2_ (red trace) and QD_n_@SiO_2_ (blue trace).

The changes in the QD@SiO_2_ nanostructure geometry, induced by TEOS concentration, only slightly affect the PL quantum yield (QY). Compared to the bare QDs, whose PL QY is 18%, a partial decrease in PL QY is observed in the silica coated samples, although a higher relative PL QY has been calculated for QD@SiO_2_ obtained with larger volume of TEOS (11%) with respect to QD_n_@SiO_2_, with PL QY of 10%. To better rationalize these results, PL recombination dynamics of as-synthesized QDs (Figure 2 black trace), QDn@SiO_2_ (Figure 2 blue trace, corresponding to QD@SiO_2__20 sample) and QD@SiO_2_ (Figure 2 red trace, corresponding to QD@SiO_2__50 sample), have been measured. The decays can be well fitted with a typical three exponential function and the average lifetimes have been calculated (table in Figure 2). The data show that the PL decay of the SiO_2_ coated QDs is faster than that observed for the as-synthesized QDs. Such a more rapid recombination can be ascribed to ligand exchange reactions caused by the SiO_2_ shell growth [12], that lowers QD passivation, increasing defect surface states, which can act as recombination centers for charge carriers, decreasing the average lifetimes. In addition, the presence of the silica shell brings a modification in the external dielectric constant experienced by the QDs and a corresponding redistribution of the energetic levels (as confirmed by the slight red shift of PL in Figure 1(E)).

The faster charge carrier recombination measured in presence of multicores QD_n_@SiO_2_ (Figure 2, blue line) can be accounted by energy transfer mechanisms among luminescent QDs embedded in the same silica shell, which are possible due to the very short inter-particle distance.

The spectroscopic and morphologic investigation clearly indicates that the nanostructures with a single QD in the core, presenting a high monodispersity, a very uniform silica shell and a reasonable PL QY, are the best candidate for luminescent nanoprobes to be used as scaffold for further decoration with the NIR absorbing Au NPs.

Indeed the silica shell with a thickness of 16 nm is expected to be large enough to avoid energy transfer processes from the photoexcited QD to the metal NPs and hence quenching phenomena.

### 3.2. Functionalization of QD@SiO2 NPs with amine groups

Primary amine groups (NH_2_) have been grafted on the silanized surface of QD@SiO_2_ (NH_2_-functionalized QD@SiO_2_ NPs) by reaction with 20, 80 and 120 μl APS in alcoholic solution under basic pH. Titration of the NH_2_ groups has been performed by means of the assay reported in [37], based on the selective reaction of ninhydrin with NH_2_ groups in the presence of 2,6-lutidine. The formation of Ruhemann’s Blue as a byproduct (see Supplementary Material Figure SI2), with its characteristic intense absorption band in the visible (λ_max_ = 570 nm) allows us to assess the presence and concentration of NH_2_ groups in the sample. From the ninhydrin test using the Lambert–Beer equation the concentration of NH_2_ groups in the final volume of 1.5 ml of suspension (containing approximately 0.4·10^15^ NPs) has been determined as nearly 7 and 10 mM for 20 and 80 μl APS, respectively. Further increase in the APS volume used for the functionalization reaction did not induce a remarkable increase in the amine group concentration, highlighting that an almost complete surface saturation has been accomplished by using 80 μl APS [20], with a density of nearly 5 NH_2_ groups nm^–2^.

ζ-potential measurements have been also carried out to investigate the change in the surface charge of the NPs upon functionalization with APS [16]. The negative ζ-potential value of the QD@SiO_2_ NPs (–37.3 ± 0.3 mV), ascribable to partially ionized silanol groups on the surface, becomes positive (+45 ± 0.1 mV) upon surface saturation with NH_2_ groups. This high ζ-potential value also indicates a very good colloidal stability of amine grafted luminescent silica coated NPs.

DLS analysis has clearly pointed out, for each step, a monomodal size distribution, thus suggesting that no NP aggregation occurs in aqueous medium and no significant increase of the average diameter passing from the QD@SiO_2_ NPs to the amine grafted QD@SiO_2_ NPs (see Supplementary Material Figure SI4). In particular, average diameter of 41.9 ± 0.9 (PDI 0.18 ± 0.02), 42.7 ± 0.6 (PDI 0.20 ± 0.02) has been recorded for the two nanostructures, respectively.

### 3.3. Decoration of amino-functionalized QD@SiO_2_ with Au nanoparticles

The positively charged NH_2_-functionalized QD@SiO_2_ NPs have been mixed with the negatively charged hydrophilic THPC-stabilized Au NPs [44, 45].

**Figure 3.**
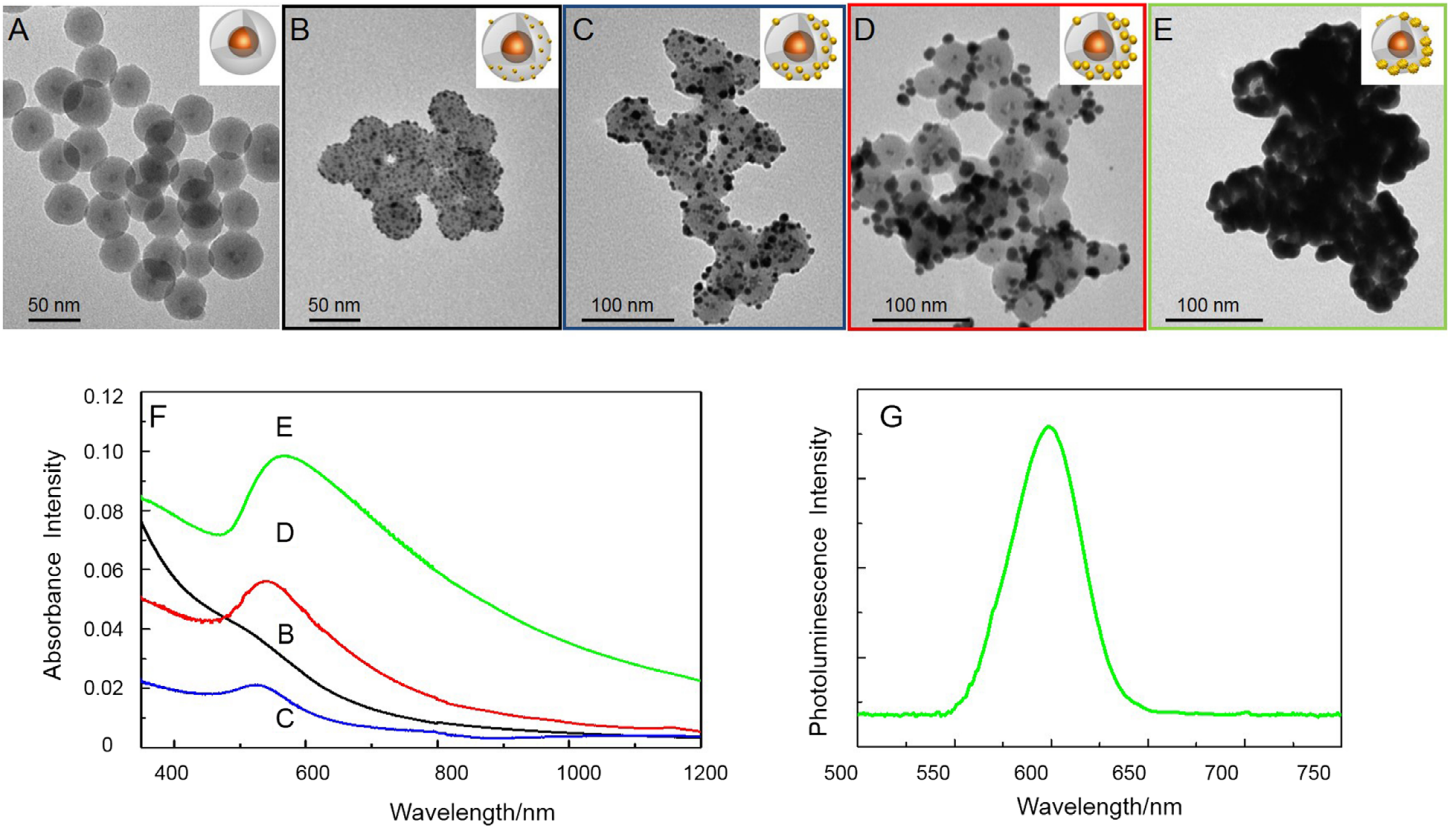
TEM micrographs of amino-functionalized QD@SiO2 before (A), and after (B) assembly of Au seeds, and (C–E) further Au deposition; each frame shows the nanoshell at different concentrations of Au precursor solutions: 0.5 M (C), 2 M (D) and 4 M (E) The UV-vis-NIR absorbance spectra of samples C–E are reported in panel F (each suspension has been diluted 1:5 in order to reduce scattering contribution to the absorption). Emission spectra of sample in Figure 3(E) (λ_ex_=400 nm).

Figure 3(A) and (B) shows the TEM micrographs of the NH_2_-functionalized QD@SiO_2_ NPs before (A) and after (B) functionalization with the Au seeds. The dark spots at the surface of SiO_2_ in Figure 3(B) can be clearly ascribed to the Au NPs assembled onto the surface of NH_2_-functionalized QD@SiO_2_ NPs (Figure 3(A)), and a surface coverage of 20–30% has been calculated on the base of TEM investigation, in agreement with previously reported studies [46, 47]. Non-specific adsorption was demonstrated to be absent (see Supplementary Material Figure SI5).

In Figure 3(F), the UV-vis absorbance spectra of the Au-decorated QD@SiO_2_ (black line) show a featureless band, that can be ascribed to very small Au NPs [46]. PL spectrum and time-resolved fluorescence intensity decays (see Supplementary Data Figure SI6) show that decoration of the QD@SiO_2_ surface with Au NPs does not significantly affect the luminescence signal. The average lifetime values (see Supplementary Data Figure SI6) of the bare QD@SiO_2_ (11.0 ns ± 0.3) remain almost unchanged after functionalization with APS and subsequent decoration with Au NPs (10.0 ns ± 0.2), thus allowing us to exclude any energy transfer phenomena between the metal and the QDs. This evidence is consistent with the fact that 16 nm thick silica shell of the QD@SiO_2_ sample is thick enough to prevent any significant energy transfer phenomena [16–18].

In order to induce isotropic Au growth on the single Au seed particle, the functionalized QD@SiO_2_ NPs have been successively mixed with suitable quantities of Au precursor, HAuCl_4_ and of K_2_CO_3_ and formaldehyde as reducing agents, in order to evaluate the influence of the molar content of the Au precursor on the final nanostructure. In Figure 3, the vis-NIR absorption spectra and the morphology of these heterostructures at increasing K_2_CO_3_/HAuCl_4_ are reported. At K_2_CO_3_/HAuCl_4_ concentrations of 0.2 mM/0.05 mM, the localized surface plasmon resonance (LSPR) peak is broad and centered at 526 nm (Figure 3(F) blue line).

The LSPR broadening can be ascribed to the large size distribution of Au islands, as confirmed by the corresponding TEM micrograph.

By increasing the Au precursor concentration the size of the Au islands progressively increases, resulting in larger and larger particles, formed by partially merging neighbor Au nanodomains onto the silica surface (Figure 3(C)). At the precursor concentrations of 0.9 mM/0.2 mM (K_2_CO_3_/HAuCl_4_), the LSPR presents a red-shift (Figure 3(F) red line), as a consequence of the further growth of the Au domains. Indeed, the corresponding TEM micrograph shows a larger Au NP coverage of silica surface (Figure 3(D)). Finally, for a K_2_CO_3_/HAuCl_4_ concentration ratio of 1.8 mM/0.4 mM, the LSPR appeared as a broaden peak at 569 nm (Figure 3(F) green line). The modifications of the LSPR band are clear indications of Au nanostructure growth [46], that results in the formation of first an incomplete (Figure 3(E)), and then a complete (see Figure SI6 Supplementary Material) Au shell, deriving from the coalescence between neighbor Au domains, as demonstrated by the corresponding TEM micrographs. Interestingly, the whole range of the investigated precursor concentrations results in NPs with a partial Au domain coverage, hence incomplete shells. Such Au domains growth with increasing concentrations of K_2_CO_3_ and HAuCl_4_. The LSPR positions measured for the prepared samples are in good agreement with previous findings on analogous systems, based on Au NPs growth on 30 nm SiO_2_ particles [46].

The sample reported in Figure 3(E) clearly shows a NIR absorbance (green line spectrum in Figure 3(F)) and a retained luminescence of the QDs. Although a partial quenching has been observed and a final PL QY of 5% has been achieved (Figure 3(G)), the obtained nanostructures can still be used as effective luminescent probes for bioimaging. The not negligible absorbance in the biologically invisible region typically used for photothermal cancer therapy (i.e. laser excitation at 808 nm) makes these nanostructures appealing to tackle the treatment of cancer cells by converting the excited state photon energy into heat.

Further growth of a complete and thick Au nanoshell brings the absorption of the nanostructure to change into a well-defined plasmon band, with no absorption tail in the NIR region, as reported [19] (Supplementary Data Figure SI7), and the emission completely disappears.

A hybrid system based on metallic and fluorescent structures has been extensively studied and PL quenching or enhancement has been observed depending on the distance and the orientation of the fluorophore with respect to the metal surface as well as on the topology of the metal surface. The Purcell effect has mainly accounted for the fluorescence enhancement arising from the plasmon and exciton coupling in nanostructures based on a continuous shell or ring of metallic materials close to a fluorophore, behaving as a metallic resonant cavity [18]. Conversely, quenching can result from charge transfer to the metal or to energy transfer and dissipation. The partial PL quenching of the here-described Au speckled nanostructures can be ascribed to the screening effect of the Au nanodomains, that absorb the excitation radiation and induce a reduction of the number of photons that reaches the QD cores, rather than being caused by interaction between metal and luminescent NPs. Due to the thickness of the silica shell, energy transfer phenomena between the metal and the QDs can be excluded in the final nanostructures, as demonstrated by the PL time-resolved characterization that shows PL decay (and average lifetime) of the NPs to be almost unchanged after decoration with Au NPs (see Supplementary Data Figure S6). On the other hand, PL enhancement due to the Purcell effect is not expected due to the size regime, geometry and morphology of the Au nanodomain decorated silica coated QD nanostructures, that, for the discontinue nature of the gold shell, is far to behave as a plasmonic fluorescent resonator.

**Figure 4.**
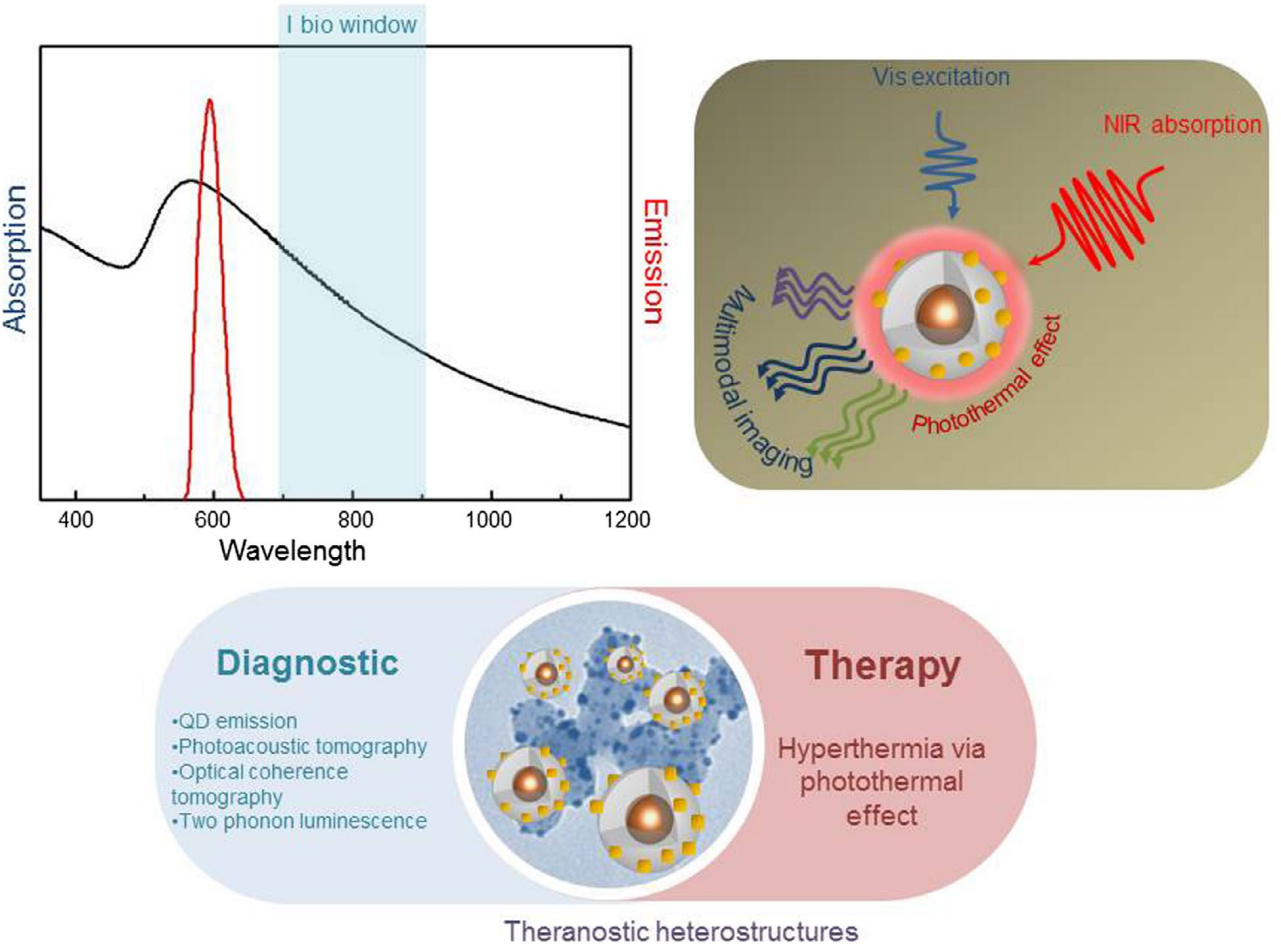
Summary of the spectral features of the obtained Au speckled QDs, which make the multifunctional architectures ideal candidate for theranostic application.

The reported data highlight that a careful control of the fabrication route of water dispersible Au-decorated QD@SiO_2_ NPs is strictly required when absorption in the NIR ‘biological tissue transparency window’ and luminescence in the visible range need to be concomitantly conveyed in the same nanostructured object (Figure 4) having a size of a few tens of nanometers. DLS investigation performed on sample E has revealed a monomodal size distribution and an average diameter of 50.6 ± 0.5 nm (PDI 0.21 ± 0.03), highlighting an increase of size passing from the amine grafted QD@SiO_2_ NPs to Au decorated nanostructures, as expected (see Supplementary Material Figure SI4). Finally, ζ-potential measurements have provided a slightly less positive value (+20 ± 0.2 mV) of Au decorated NPs with respect to that of the amino functionalized nanostructures, which can be ascribed to the decrease of the free amino groups present at the surface of Au-decorated QD@SiO_2_ NPs, due to their partial neutralization by the growth of metal nanodomains.

In order to test the viability of the prepared Au-decorated QD@SiO_2_ nanostructures in biologically relevant *in vitro* studies, their cytotoxicity response has been investigated, by testing the nanomaterials at the different stages of their processing.

For this purpose *in vitro* growth inhibition of rat C6 rat glioma cells and MTT assay have been used for the cytotoxicity assessment, as C6 rat glioma cells represent an experimental model system for glioblastoma multiforme, having been also widely studied for elucidating mechanism of tumor growth, angiogenesis and invasion, and in design and evaluation of anti-cancer therapies [47].

In particular, cytotoxicity has been tested for a long (72 h) incubation period, exploring NP concentrations ranging from 0.1 mg ml^–1^ to 0.001 mg ml^–1^. In Table 1, IC_50_ values for QD@SiO_2_, APS-functionalized QD@SiO_2_ and Au decorated QD@SiO_2_ have been reported.

**Table 1.** *In vitro* growth inhibition of rat C6 glioma cells treated with the different types of nanostructure at increasing concentrations.

Nanomaterial sample	IC_50_ (μg ml^–1^)^a^
QD@SiO_2_	30.1 ± 0.9
NH_2_-functionalized QD@SiO_2_	9.2 ± 0.6
Au NPs-decorated QD@SiO_2_	13.5 ± 0.8

^a^ Data are presented as mean ± standard error of three separate experiments performed in triplicate.

The obtained results indicate that functionalization of QD@SiO_2_ NPs with APS causes a decrease in cell vitality (IC_50_ = 9 μg ml^–1^), reasonably ascribed to the positive charge expressed by the NP surface.

These results are in good agreement with those previously observed for other positively charged NPs [48], that show a higher cytotoxicity as long as the amount of amine groups on the NP surface increases. Interestingly upon functionalization with Au NPs, the IC_50_ value slightly increases with respect to that observed for NH_2_-functionalized QD@SiO_2_ (13.5 μg ml^–1^ and 9.2 μg ml^–1^, respectively), thus suggesting that the presence of Au NPs at the QD@SiO_2_ surface enhances their biological viability and demonstrating that a suitably surface engineering procedure can control their biocompatibility.

## 4. Conclusions

A visible-NIR photoactive heterostructure based on Au nanodomain decorated luminescent QD@SiO_2_ NPs with size in the submicrometer range have been successfully fabricated by suitably tailored colloidal strategies.

Different types of heterostructure, differing in QD multiplicity inside the SiO_2_ NPs, the SiO_2_ shell thickness and extent of Au nanodomain deposition onto SiO_2_ surface, have been prepared by tuning the experimental parameters, in order to accomplish a nanostructure whose optical properties fit with the aims of the work. The deep morphological and optical characterization shows that a multifunctional heterostructure able to preserve the QD emission in the visible region and to generate NIR absorption from metal nanodomains has been achieved.

The synthesized nanostructure represents one of the few examples of all inorganic colloidal NPs which combines in a submicrometer sized heterostructure the QD luminescence and Au NIR absorption.

The good photostability of QDs, the high stability in aqueous solution and their biological viability make these multifunctional heterostructures a promising nanoplatform highly relevant for potential applications in nanobiotechnology, as multimodal bioimaging agents and as mediators of photothermal therapy.

## Disclosure statement

No potential conflict of interest was reported by the authors.

## Supplementary Material

Supplementary Data
