# Supplementary material for: Fabrication of photoactive heterostructures based on quantum dots decorated with Au nanoparticles
Source: Sci Technol Adv Mater. 2016 Apr 12;17(1):98–108. doi: 10.1080/14686996.2016.1153939 (PMC5101891; doi:10.1080/14686996.2016.1153939)
Supplement: Supplementary Data [file tsta_a_1153939_sm8444.docx]

**Supplementary Data**

**Fabrication of Photoactive Heterostructures based on Quantum Dots Decorated with Au Nanoparticles**

Elisabetta Fanizza^1,2^, Carmine Urso^1, †^, R. Maria Iacobazzi^3,4^, Nicoletta Depalo^2^, Michela Corricelli^1,2^, Annamaria Panniello^2^, Angela Agostiano^1,2^, Nunzio Denora^3^, Valentino Laquintana^3^, Marinella Striccoli^2^ and M. Lucia Curri^2^

^1^ Dipartimento di Chimica, Università degli Studi di Bari, Via Orabona 4, 70126 Bari, Italy.

^2^ Istituto per i Processi Chimico Fisici IPCF Consiglio Nazionale delle Ricerche CNR, Via Orabona 4, 70126 Bari, Italy.

^3^ Dipartimento di Farmacia – Scienze del Farmaco, Università degli Studi di Bari, Via Orabona 4, 70126 Bari, Italy.

^4^ Istituto tumori IRCCS Giovanni Paolo II, Bari, Italy.

† present address: Dipartimento di Chimica e Chimica Industriale, Università degli Studi di Genova, via Balbi 5, 16126 Genova; IIT Via Morego 30, 16163 Genova

Corresponding Author e-mail: [elisabetta.fanizza@uniba.it](mailto:elisabetta.fanizza@uniba.it) and [n.depalo@ba.ipcf.cnr.it](mailto:n.depalo@ba.ipcf.cnr.it)

**Synthesis of CdSe@ZnS QDs.** Core-shell structures CdSe@ZnS QDs have been synthesized following a reported approach with minor modification (W. W. Yu, L. Qu, W. Guo, X. Peng, Chem. Mater. 15 (2003) 2854). The synthesis is based on a multistep procedures consisting in a hot injection method for the synthesis of the core followed by the drop-wise injection of ZnS precursor solution in the same reaction flask to promote the formation of the ZnS shell. In particular, for the core synthesis 0.127 g of CdO, HDA (12 g), TOPO (12 g) and ^t^BuPOH (0.276 g) in a three-neck flask have been warmed up under inert atmosphere and degassed for 1h. The solution temperature has been then increased up to 290°C under vigorously stirring and the red coloured mixture turns colourless due to the decomposition of CdO. The temperature has been then set to 300°C, the Se/TBP (0.394 g /4.40 g) injected which causes the formation of the first CdSe nuclei. The core growth has been carried out at 270°C keeping the temperature constant till the colloidal nanocrystals reached the suitable size. The longer the reaction time the bigger the particle. The core growth has been here carried out for 5 min (resulting in a core size of 4.2 nm), then the reaction mixture has been cooled down to 100°C to stop the growth of the QDs. For the shell growth a stock solution of ZnS precursors containing TBP (9.46 g), Et_2_Zn (1.294 g) and HMST (0.38 g) , prepared in glove box, has been drop-wise injected in the reaction flask at 155°C. Core-shell CdSe@ZnS QDs as red powder have been then collected by centrifugation after the addiction of methanol as non-solvent and dispersed in a suitable amount of chloroform or other non polar organic solvent, if required. The final QD concentration of 1.8·10^-4^ M.


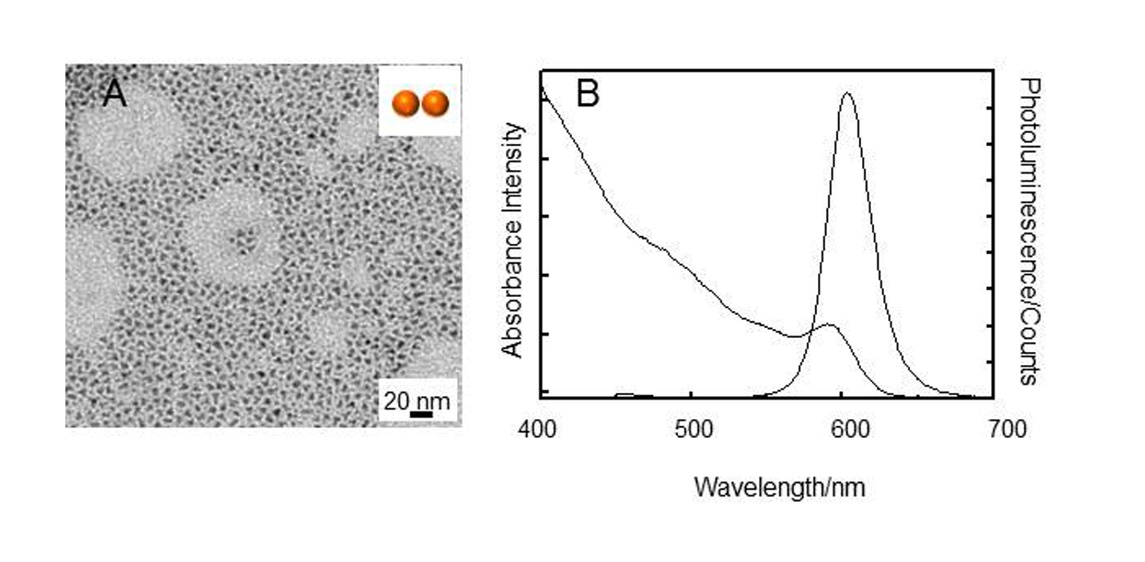


**Figure SI 1.** TEM image (A) and absorption and emission spectra of (B) of CdSe@ZnS QDs in chloroform.

In Figure SI 1 the spectroscopic and structural characterizations of the as synthesized TOPO/HDA-capped CdSe@ZnS QDs have been reported. The absorption spectrum (Figure SI 1B) of QDs shows a peak corresponding to the first excitonic transition centered at 590 nm, which corresponds to QD of about 4.2 nm, in agreement with the TEM result (Figure 1A).

The UV-Vis absorption spectral line-shape and the narrow photoluminescence (PL) line-width, (110 meV), centered at 604 nm, indicate a good monodispersity of the QDs, as confirmed by TEM statistical analysis (α%= 19%).

| **Sample** | **Lifetime (ns)** | **t1 (ns)** | **t2 (ns)** | **t3 (ns)** | **A1 (%)** | **A2 (%)** | **A3 (%)** |
| --- | --- | --- | --- | --- | --- | --- | --- |
| QD | 14 ±0.02 | 1.68±0.03 | 9.8±0.2 | 33.5±0.4 | 17.22±0.01 | 59.45±0.04 | 23.33±0.02 |
| QD@SiO_2_ | 11 ±0.03 | 1.92±0.07 | 5.0±0.3 | 16.2±0.4 | 7.79±0.02 | 37.70±0.02 | 54.51±0.05 |
| QD_n_@SiO_2_ | 8 ±0.01 | 1.37±0.02 | 3.07±0.09 | 12.7±0.2 | 9.27±0.02 | 37.80±0.05 | 52.93±0.03 |

**Table S1.** Average lifetime values, components and amplitude parameters used to fit the three exponential function decays for QDs, QD@SiO_2_ and QD_n_@SiO_2_ nanoparticles.

Water-in-oil microemulsion approach has been used to coat the CdSe@ZnS QDs with a SiO_2_ shell further functionalized with 3-Aminopropyltrimethoxysilane (APS).

In order to univocally proof the presence of surface grafted amine groups an assay, reported in literature (N. Depalo, L. Catucci, A. Mallardi, A. Corcelli, A. Agostiano, Bioelectrochemistry, 63 (2004) 10) to typically evidence primary amines groups for biomolecules, has been suitably adjusted and exploited for APS-functionalized QD@SiO_2_ NP surface. The protocol takes advantage of selective reaction of ninhydrin with primary amine groups in the presence of 2,6 lutidine. The addiction of ninhydrin/2,6 lutidine solution to primary amine results in the formation of Ruhemann's Blue by-product that has a characteristic absorbance in the visible range with an intense band centered at 570 nm (Figure SI 2A).


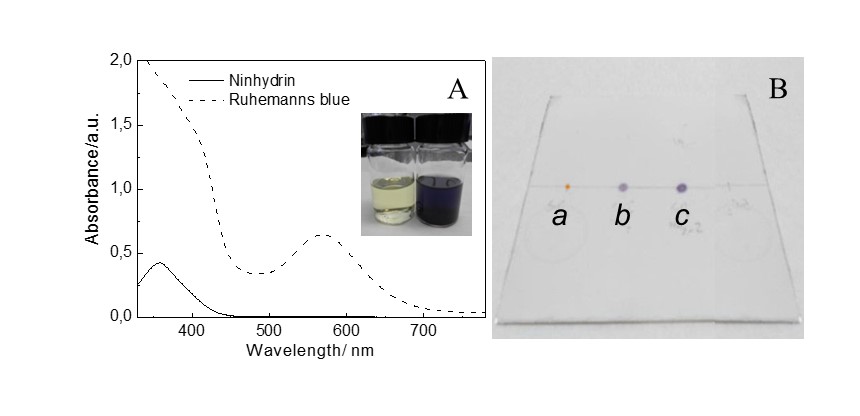


**Figure SI2**. (A) UV-Vis absorbance spectra of ninhydrin solution (straight line) and Ruhemann's Blue (dot line) and (B) TLC plate with deposited drops of QD@SiO_2_ NPs (a) and APS-functionalized QD@SiO_2_ NPs with 20μL (b) and 80 μL (c) of APS after spaying the ninhydrin/2,6 lutidine solution in acetone. Inset Figure A. Picture of the ninhydrin solution (left) and Ruhemann's Blue by-product (right).

In particular a qualitative thin layer chromatography (TLC) analysis has been carried out: a drop of the amine functionalized samples was put on the TLC plate and sprayed with the ninhydrin solution in acetone (110 mg of ninhydrin in 16 mL of acetone 0.68% w/v, and adding 4 mL of 2,6 lutidine). TLC characterization has confirmed the presence of amino groups by the formation of a blue coloured spot ascribed to the Ruhemann's by-product.

As prepared QD@SiO_2_ NPs, APS-functionalized QD@SiO_2_ NP with 20 μL (Figure SI2B *b*) and APS-functionalized QD@SiO_2_ NP with 80 μL (Figure SI2B *c*) have been deposited onto the TLC plate. The spots corresponding to APS-functionalized QD@SiO_2_ NP (Figure SI2B *b,c*) appear blue stained after spraying the ninhydrin/2,6 lutidine solution in acetone, while the QD@SiO_2_ NPs (Figure SI3B *a*) keeps the QD orange colour. The selective blue staining of the spots, with a higher colour intensity as expected from the larger volume of APS used for the reaction, provides a qualitative evidence of the presence of primary amine.

The extent of the functionalization will obviously depend from the amount of added functionalizing agent and the NP concentration ad size, that define the available surface area. For the collected APS-functionalized NPs, a loss of 10% of NPs have been estimated, as measured by blank experiments, as consequence of the purification steps, based on redispersion/centrifugation cycles.

The general approach to estimate the concentration QD@SiO_2_ relies on the measure of the NP radius as calculated from the TEM characterization, the experimentally weighted lyophilized samples (Wex_NPs_), the literature reported data of the material density, which, together with the NP size and shape, provide the theoretic weight of a single spherical beads (Wt_NP_). The total number of NPs is calculated from the ratio Wex_NPs_ /Wt_NP_.

For SiO_2_ NPs, the theoretical silica NP concentration has been calculated by taking into account the density of the SiO_2_ NP (2 g/cm^3^), the NP geometric volume obtained from the average NP radius, measured by TEM and the weight of the collected lyophilized powder. This method to evaluate the NP concentration agreed with the approach reported in literature, that assume 1 mole of TEOS precursor to generate 34 cm^3^ of SiO_2_ NP. (A. van Blaaderen, D. Vanmaekelbergh, A. Meijerink, Chem. Mater., 20 (2008) 2503)

A similar method has been here developed to estimate the concentration of QD@SiO_2_. It takes into account (i) the density of SiO_2_ and CdSe (5.8 g/cm^3^, the contribution of the ZnS is neglected due to the very thin thickness of the ZnS shell in the CdSe@ZnS nanostructure), (ii) the radius of QD@SiO_2_ as measured by the TEM investigation (iii) the experimentally weighted powder collected from the lyophilized sample (Wex_NPs_=0.005 g/200μL). Using the data reported in the table in Figure SI3, the geometric calculation and the experimentally determined weight of the lyophilized sample, the number of QD@ SiO_2_ can be obtained, which has resulted 10^15^ NPs in 4 mL, in agreement with the QD concentration and the single QD core per bead geometry of the sample.


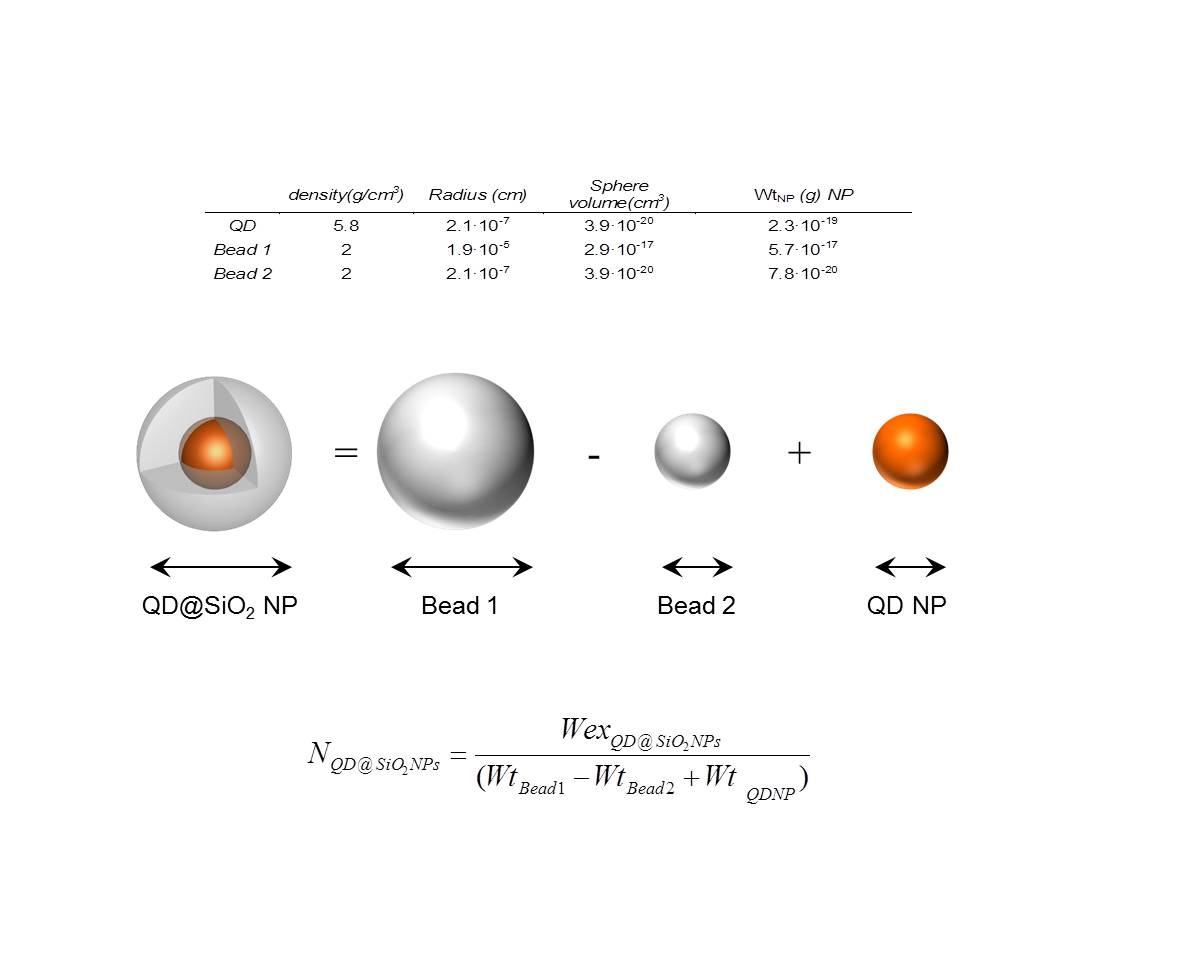


**Figure SI3**. Scheme, table and equation to calculate the QD@SiO_2_ concentration.

**
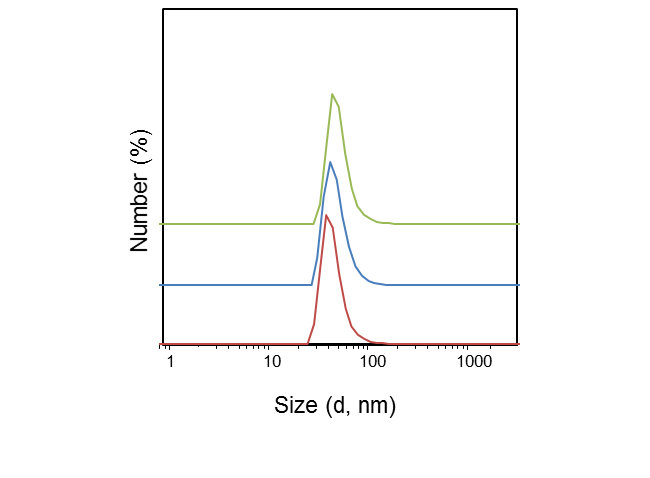
**

**Figure SI 4.** Size distribution by dynamic light scattering measurements of QD@SiO_2_ (red line), NH_2_-functionalized QD@SiO_2_ (blue line) and Au-decorated QD@SiO_2_ (green line)

**Gold nanoshell formation onto QD@SiO_2_.** TEM micrograph of unfunctionalized QD@SiO_2_ samples after mixing with THPC stabilized- Au NPs are reported in Figure SI5, reveling that aspecific adsorption of Au NPs onto QD@SiO_2_ can be undoubtedly ruled out as no dark spot ascribed to the metal NPs can be detected. THPC stabilized- Au NPs have been assembled onto NH_2_-functionalized QD@SiO_2_ (Figure SI6). This results confirms that the key role of NH_2_ moieties to guide the deposition of Au onto the silica surface.


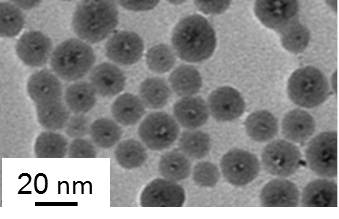


**Figure SI5.** TEM image of QD@SiO_2_ upon incubation with THPC-stabilized Au NPs.


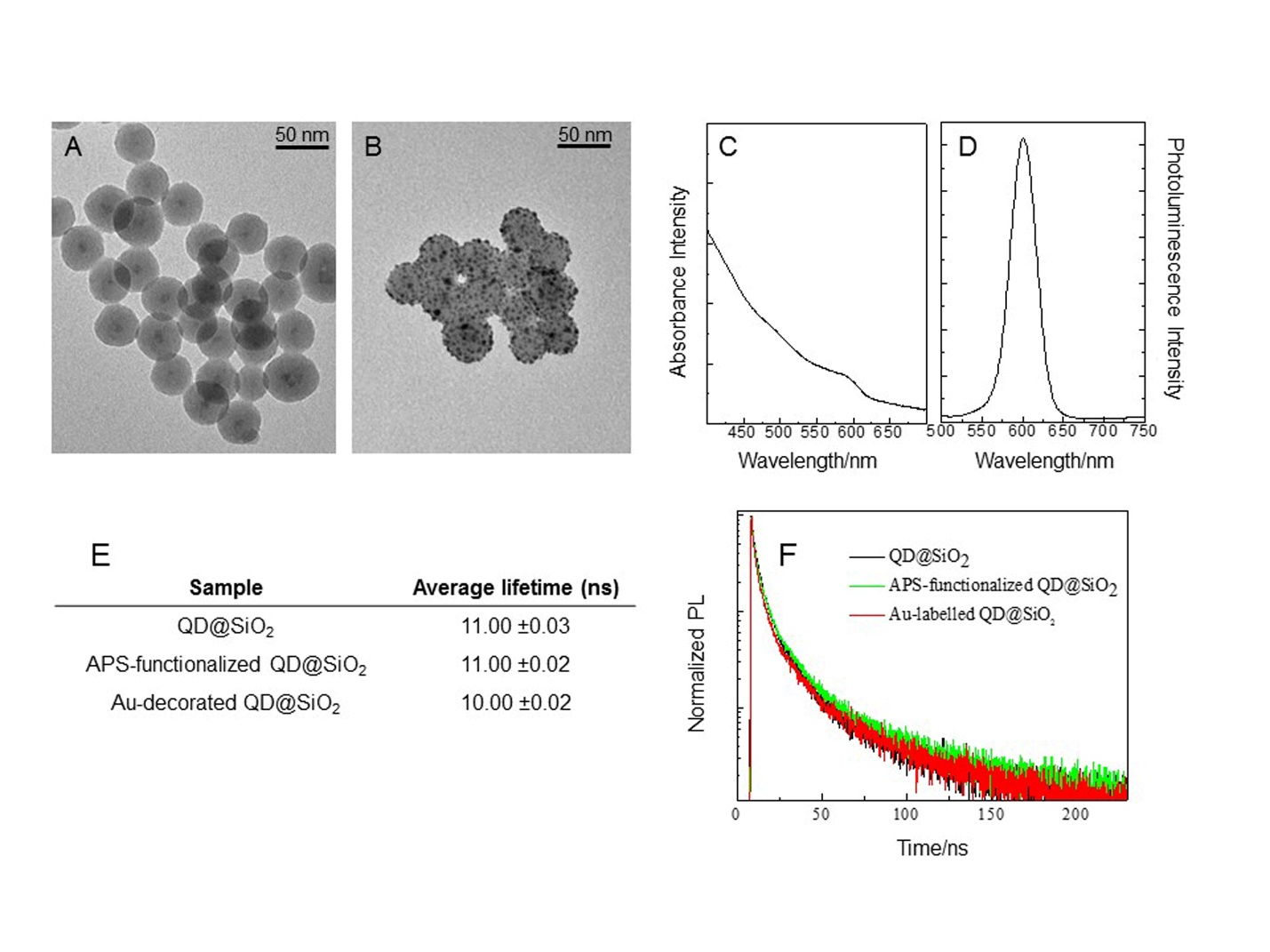


**Figure SI6.** TEM micrographs of APS-functionalized QD@SiO_2_ before (A) and after (B) assembly of Au seed. UV-Vis Absorbance (C) and PL (D) spectra of the Au-speckled QD@SiO_2_. Time-resolved fluorescence intensity decays (F) and average lifetime (E) of QD@SiO_2_, APS-functionalized QD@SiO_2_ and Au decorated QD@SiO_2_.


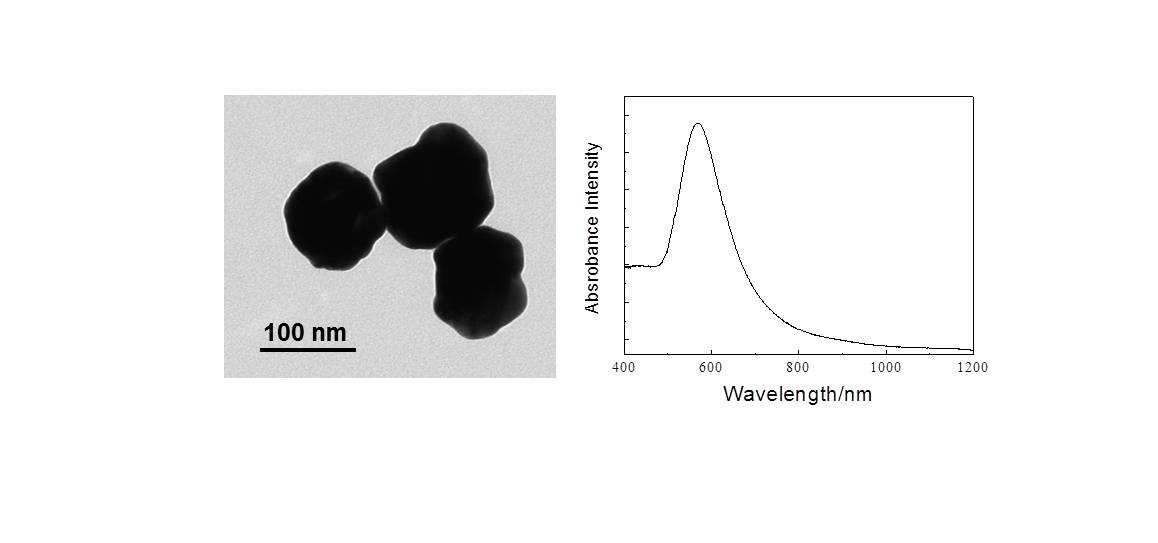


**Figure SI7.** TEM image and Vis-NIR absorbance spectrum of a sample with nanoparticles with a complete Au nanoshell onto QD@SiO_2_.
